# The preliminary application of electromyography in unilateral biportal endoscopy with general anesthesia for lumbar disc herniation

**DOI:** 10.3389/fsurg.2025.1498878

**Published:** 2025-05-27

**Authors:** Shaolong Tang, Yutian Liao, Juan Pan, Dayong Chen, Dan Pan

**Affiliations:** ^1^ Department of Spinal Surgery, Zhuzhou Central Hospital, Zhuzhou, Hunan, China; ^2^ Department of Trauma, Zhuzhou Central Hospital, Zhuzhou, Hunan, China; ^3^ Department of Ultersound, Zhuzhou Central Hospital, Zhuzhou, Hunan, China

**Keywords:** discectomy, unilateral biportal endoscopy, minimally invasive surgery, electromyography, general anesthesia

## Abstract

**Objective:**

To investigate the clinical efficacy of electromyography (EMG) in unilateral biportal endoscopy (UBE) with general anesthesia in the treatment of lumbar disc herniation.

**Methods:**

A total of 78 patients with lumbar disc herniation were enrolled. They underwent UBE discectomy under general anesthesia, with the entire procedure of EMG monitoring. Recorded potentials were displayed on the monitoring screen, and electromyographic activity was audibly relayed via speakers. Clinical treatment outcomes were assessed using Visual Analog Scale (VAS) and Oswestry Disability Index (ODI).

**Results:**

All 78 patients successfully completed the procedure, with significant improvement in symptoms postoperatively. Preoperative and 3-month postoperative VAS scores were 4–9 (mean 6.65 ± 1.53) and 0–4 (mean 1.40 ± 1.23), respectively. Preoperative and 3-month postoperative ODI scores were 36–88 (mean 59.56 ± 13.81) and 4–29 (mean 14.82 ± 6.68), respectively, with statistically significant differences (*P* < 0.05). Abnormal EMG changes, including spike, burst, or tonic electromyographic discharges, occurred in 12 patients during surgery, with an incidence of 15.38%. Ten patients experienced radicular burning pain and abnormal lower limb sensations postoperatively, while two patients had no significant postoperative neuralgia, resulting in a false positive rate of 16.67%. Patients without abnormal EMG responses during surgery had no significant postoperative neuralgia, yielding a false negative rate of zero.

**Conclusion:**

General anesthesia combined with UBE monitord by intraoperative EMG is a safe and feasible approach for the treatment of lumbar disc herniation.

## 1 Introduction

Intervertebral disc herniation in the lumbar spine is the most common degenerative condition, characterized by lumbar pain and radiating pain in the lower extremities. The primary approach to treatment is conservative management, encompassing pharmacotherapy, physical therapy, and neural blockade procedures. Surgical intervention may be considered for patients experiencing severe neurogenic pain and mobility impairment refractory to conservative treatment. Unilateral biportal endoscopy (UBE) decompression surgery has emerged as an effective strategy for improving neurogenic pain and enhancing the quality of life in such patients. UBE minimizes damage to the posterior muscle and ligamentous structures, preventing postoperative segmental instability. In recent years, UBE has gained increasing prominence in the surgical management of lumbar disc herniation. As technology has evolved, it has also been extended to procedures such as lumbar spinal stenosis decompression, posterior cervical decompression, and lumbar fusion (1, 2).

UBE procedures require general anesthesia, and during surgery, the operator lacks real-time monitoring of the patient's lower limb pain and mobility. This limitation contributes to potential risks of neural injury. Postoperative radicular pain and abnormal lower limb sensations are the most common complications following lumbar endoscopic surgery, with incidence rates ranging from 7% to 25% (3). These complications are predominantly associated with excessive traction or compression of neural roots during surgery and the excessive use of bipolar radiofrequency electrocautery, resulting in pronounced neural congestion and edema. Intraoperative neurophysiological monitoring is commonly employed in cervical and thoracic spine surgeries (4, 5). However, it is less commonly utilized in lumbar spine surgery due to the ability to directly visualize the nerves and prevent injury during the procedure. With the advancement of minimally invasive lumbar surgical techniques, intraoperative neurophysiological monitoring can offer an added layer of safety in situations with limited visualization. Currently, there is limited literature reporting the application of intraoperative neurophysiological monitoring in the context of UBE.

This study included 78 patients with lumbar disc herniation who underwent UBE surgery at our institution from January 1st 2020 to January 1st 2023. The entire surgical procedure was performed under continuous monitoring of free-run electromyography (fEMG). Clinical treatment outcomes were assessed using the Visual Analogue Scale (VAS) and Oswestry Disability Index (ODI). The results of the study are summarized as follows.

## 2 Materials and methods

### 2.1 General data

Inclusion criteria: (1) A definite diagnosis of lumbar disc herniation, with significant radiating pain and/or numbness of lower limbs; (2) The imaging findings of CT and MRI of lumbar spine are consistent with medical history and physical examination; (3) Conservative treatment for more than 3 months is ineffective; (4) Follow-up for more than 12 months.

Exclusion criteria: (1) Previous history of lumbar surgery; (2) Lumbar infection, tumor, trauma, deformity, instability;(3) Combined with serious mental disorders; (4) Patients who cannot tolerate general anesthesia surgery.

78 cases were included, 38 males and 40 females. Age: 22–63 (41.44 ± 12.93) years old. Herniated disc segments: L2/3 (4 cases), L3/4 (7 cases), L4/5 (31 cases), L5/S1 (36 cases). Types of lumbar disc herniation: herniated type in 21,ruptured type 21 and prolapsed type 36. The medical history was 3–18 (8.67 ± 3.60) months. Preoperative VAS and ODI were 4–9 (6.65 ± 1.53) and 36–88 (59.56 ± 13.81), respectively.

This study has been approved by the Ethics Committee of Zhuzhou Central Hospital of Hunan Province, China. All patients informed the study risks and benefits and signed the informed consent form.

### 2.2 Anesthesia and surgical approach

Total intravenous anesthesia was administered using propofol (4–8 mg/kg/h) and remifentanil (0.3–0.8 mg/kg/min), with muscle relaxants employed solely for intubation. Following the induction of satisfactory general anesthesia, the patient was positioned in a flexed hip and knee posture. The responsible disc level was identified under fluoroscopy, with the location marked on the patient's skin. The upper border of the superior facet joint of the affected side vertebrae and the lower border of the inferior facet joint of the adjacent vertebrae converged at the transition zone between the spinous process and lamina. After confirming the position using fluoroscopy, a sequential dilator was used to create a working corridor by blunt dissection after the dilator reached the puncture sheath of the endoscope. Following endoscope insertion, the lower border of the superior lamina was identified, and the laminar window was exposed. Partial laminectomy was performed on the lamina under endoscopic guidance using a drill and rongeur. The ligamenta flava was incised at the medial edge of the facet joint under endoscopic visualization to access the spinal canal. Once inside the spinal canal, the exiting nerve root's lateral edge was exposed, and a nerve hook was used to gently retract the nerve root toward the midline, revealing the protruding intervertebral disc. The protruding disc was removed, and thorough neural decompression and hemostasis were achieved. The two incisions were then closed with a full-thickness suture using 2–0 antimicrobial nylon thread at two locations.

### 2.3 Intraoperative neurophysiological monitoring methods and principles

All monitoring procedures were conducted by the same neurophysiologist. Electromyographic (EMG) recordings were obtained from the bilateral quadriceps femoris muscles (L3, if necessary), adductor longus muscle (L4), tibialis anterior or fibularis longus muscles (L5), and gastrocnemius muscle (S1). The recorded potentials were displayed on the monitoring screen and played through a speaker for the surgical team to hear the muscle activity. Consistently appearing atypical waveform discharges, substantial amplitude changes, or bursts of activity were considered abnormal indicators. Surgeons were required to promptly halt the procedure when such abnormalities occurred, investigate the cause of the neural anomaly, adjust the surgical approach, and avoid neural damage that might lead to adverse consequences. Motor evoked potentials were elicited before and after the surgery, with alarm criteria defined as: 1. A greater than 50% decrease in amplitude compared to preoperative levels; 2. An increase in stimulation intensity exceeding 100 volts compared to baseline thresholds, lasting longer than 1 h without eliciting a response. The occurrence of either of these criteria was considered indicative of neural injury.

### 2.4 Postoperative management

Following the procedure, standard pain management was administered. Patients were instructed to wear a lumbar brace for a period of 3 weeks post-surgery. For the first 3 months post-surgery, patients were advised to avoid strenuous activities. Bending, heavy lifting, prolonged sitting, and strenuous physical labor were to be avoided during this period.

### 2.5 Observational parameters

All patients' intraoperative fEMG data and any occurrences of complications were documented. The clinical treatment outcomes were evaluated using the VAS and the ODI.

### 2.6 Statistical analysis

Statistical analysis was conducted using SPSS 17.0 software. Continuous variables are expressed as mean ± standard deviation ($\bar{x}\pm s$), and comparisons were made using t-tests. Categorical data were compared using chi-square tests.

## 3 Results

**Figure 1:**
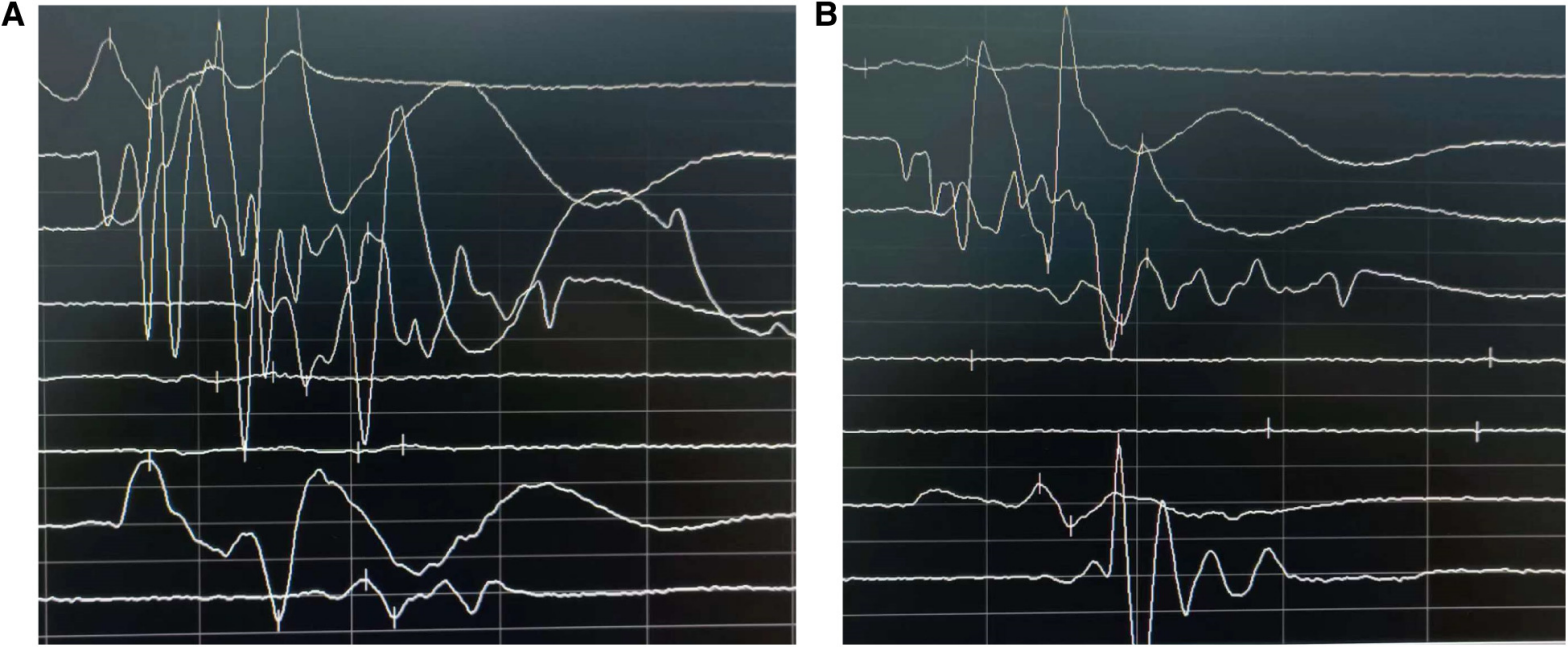
Comparison of motor evoked potentials before surgery **(A)** and after completion of surgery **(B)** postoperatively, motor evoked potentials were elicited and displayed no significant amplitude reduction.

A total of 78 patients successfully underwent surgery, motor evoked potentials were elicited and displayed no significant amplitude reduction before incision closure (Figure 1), and their symptoms significantly improved postoperatively. The VAS scores before surgery and at 3 months post-surgery were 4–9 (mean 6.65 ± 1.53) and 0–4 (mean 1.40 ± 1.23), respectively. The ODI scores before surgery and at 3 months post-surgery were 36–88 (mean 59.56 ± 13.81) and 4–29 (mean 14.82 ± 6.68), respectively. These differences were statistically significant (*P* < 0.05).

**Figure 2:**
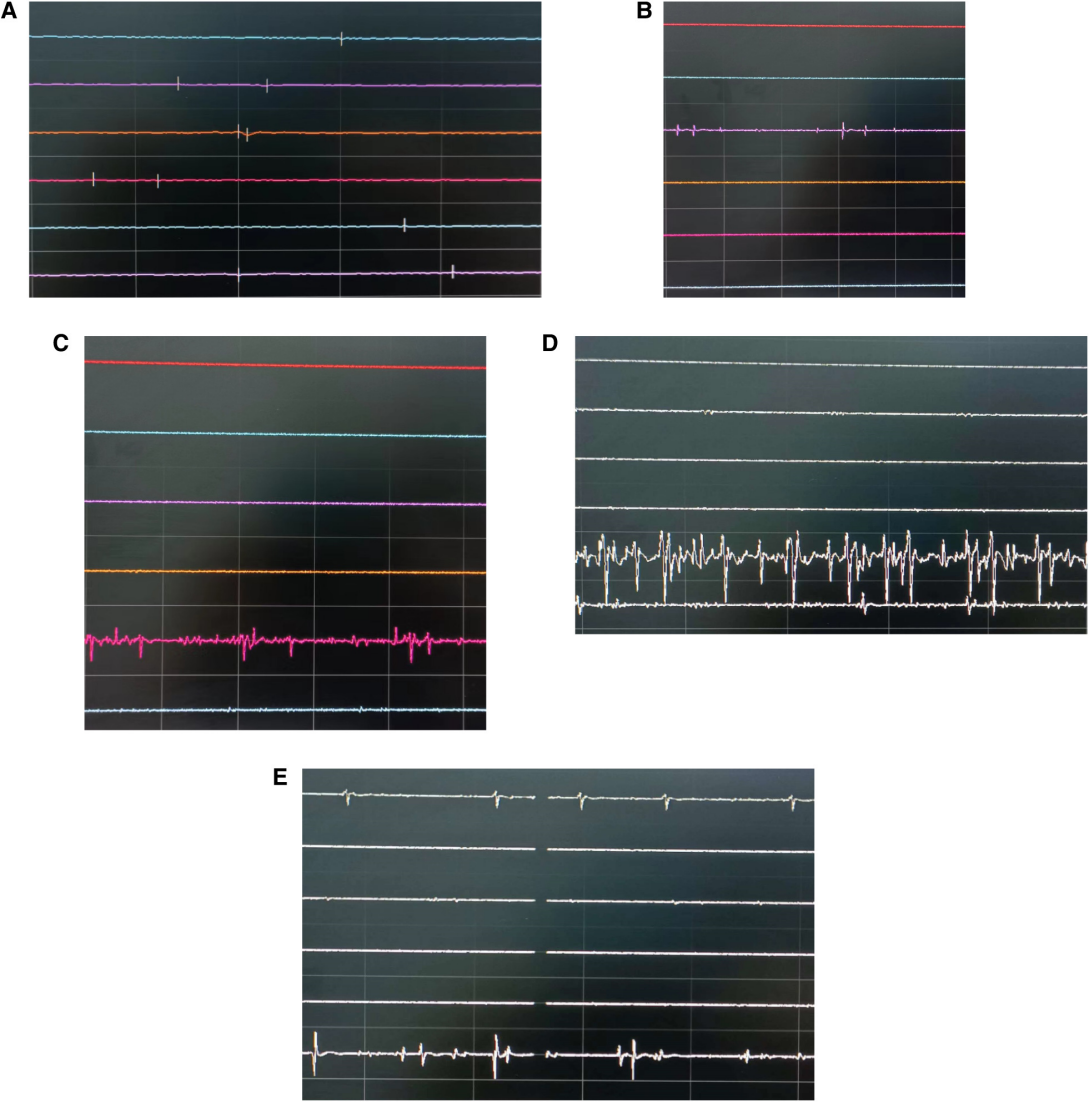
Different intraoperative electromyographic discharges (the top three lines represent three target muscles on the left side, and the bottom three lines represent three target muscles on the right side). **(A)** No obvious action potentials. **(B)** Spikes: transient appearance of sharp-wave activity. **(C)** Bursts: transient burst-like waveforms. **(D)** Tonic discharges: continuous, high-amplitude action potentials. **(E)** Burst discharges on the affected side and spike wave EMG on the healthy side.

Abnormal electromyographic changes were observed in 12 patients during surgery, with an incidence rate of 15.38%. Among them, 2 patients exhibited spike wave EMG, 4 patients had burst EMG, 3 patients had tonic EMG, and 3 patients displayed both burst and tonic EMG. Two patients experienced abnormal EMG on the healthy side. One case was attributed to an unnoticed dural sac rupture during the surgical procedure, and the other case was due to suboptimal endoscope navigation, causing the operative channel to cross the midline and stimulate the healthy-side nerve root (Figure 2). Among the 12 patients with abnormal EMG responses, 10 developed radicular pain and lower limb sensory abnormalities postoperatively but improved after conservative treatment. Two patients did not experience significant nerve pain postoperatively, resulting in a false-positive rate of 16.67%. Patients without abnormal EMG responses during surgery had no significant postoperative nerve pain, resulting in a false-negative rate of zero.

## 4 Discussion

In 2017, Heo et al. (6) first introduced the term UBE technique, and numerous studies have since demonstrated its effectiveness in treating lumbar disc herniation. The primary advantages of UBE technology include: (1) The use of conventional arthroscopic surgical instruments for the procedure. (2) The ability to have both observation and working channels without mutual interference, significantly expanding the endoscopic field of view and the workspace for surgical instruments. (3) A surgical pathway and spinal decompression process that are similar to traditional lumbar discectomy. (4) The capability to utilize various conventional open surgical instruments, enhancing operational efficiency. Neurological tissue damage is considered the most significant complication of UBE surgery, with the most common complications being dural tear and nerve root injury, with an incidence ranging from 6.6% to 13.8% (7, 8). Intraoperative neurophysiological monitoring, including somatosensory evoked potentials, motor evoked potentials, and EMG, has been applied and proven effective in preventing neurological injury during spinal surgery. There is ongoing debate regarding whether neurophysiological monitoring is necessary for lumbar surgery and which monitoring modalities should be used (9). One of the essential objectives of intraoperative neurophysiological monitoring in lumbar endoscopic surgery is to assist in identifying concealed or displaced nerve roots, assessing their function and integrity, and preventing irreversible damage (10). Somatosensory evoked potentials and motor evoked potentials are neither highly specific nor sensitive enough to assess the functionality of individual nerve roots (11). Haghighiss et al. found in their study of C5 nerve root injuries in spinal surgery that EMG sensitivity far exceeded that of somatosensory evoked potentials and motor evoked potentials (12).

The reflex activity of spinal nerves represents one of the most complete and sensitive functions of the nervous system, relying on the synchronous conduction of incoming and outgoing nerve fibers. Unlike peripheral nerves, spinal nerve roots lack perineurium and are covered only by a thin epineurium. Nerve roots do not have segmental vascular supply but rely on nerve root arteries (13). Preoperatively, due to compression of the nerve roots by intervertebral disc herniation, nerve roots become more susceptible to additional mechanical loads. Traction of nerve roots by as little as 3 mm (approximately 70 g/cm^2^ of pressure) during surgery can reduce nerve blood supply to 20% of its initial value. Further increases in mechanical load can lead to segmental demyelination, conduction block, and decreased action potential amplitudes. Identifying nerve function impairment in the reversible stage during surgery can help prevent permanent damage (14).

fEMG mainly records the motor units obtained from the muscles innervated by specific nerves, and these motor units are related to the depolarization that occurs when the nerve is stimulated by stretching, compression, clamping, etc. The more motor units recorded and the more obvious they are, the greater the intensity of the nerve stimulation. Therefore, fEMG monitoring can continuously and dynamically reflect the status of the target nerve root during surgery, thereby effectively reducing the incidence of nerve injury complications.

In this study, two patients exhibited spike wave EMG, characterized by single asynchronous potentials, and the transient appearance of sharp peaks. Seven patients displayed burst EMG, which featured a complex multiphase structure and transient burst-like waveforms. Spike wave EMG or burst EMG is associated with contact with nerve roots, such as compression or traction of the nerve roots (15). During UBE surgery, when dissecting the ligamenta flava of the hidden side foraminas or retracting nerve roots for nucleus removal, contact between neural tissue and surgical instruments can trigger burst EMG. Two patients experienced abnormal EMG responses on the healthy side. In one case, it was due to an unnoticed dural sac rupture during surgery, which directly affected the healthy-side nerve root due to increased water pressure, resulting in abnormal EMG responses. In the other case, suboptimal endoscope navigation led to the operative channel crossing the midline and stimulating the healthy-side nerve root. Therefore, this method exhibits a sufficiently high sensitivity to identify neural structures at the surgical site. To validate these findings, more extensive investigations are required in larger patient populations.

In six cases, patients exhibited tonic discharges during surgery, characterized by prolonged, repetitive synchronous bursts of motor unit discharges that could last for several minutes. This type of EMG activity is typically associated with continuous traction or compression of nerve roots (15, 16). It indicates a more severe pathological response and should be considered a warning sign of nerve injury. In all six cases, the surgeons responded immediately by repositioning instruments, and no postoperative deterioration in motor function was observed. It is noteworthy that all six patients experienced radicular pain and lower limb sensory abnormalities postoperatively, suggesting more severe mechanical stimulation of the nerve roots. Their symptoms improved after bed rest and symptomatic management, including neurotrophic therapy.

During surgery, fEMG should distinguish between changes caused by the surgical procedure and those induced by anesthesia. fEMG can only be recorded during surgery when anesthesia does not suppress the activity of spinal interneurons and alpha motor neurons (17). Most anesthetic agents reduce the amplitude and increase the latency of electrophysiological potentials. In previous studies, total intravenous anesthesia had little to no impact on electromyographic monitoring (18). Therefore, all 78 cases reported in this study were conducted under total intravenous anesthesia. Short-acting muscle relaxants (such as rocuronium) were used solely for intubation, and normal signals were recorded only after a recovery period of 15–20 min.

Discharges not related to muscle activity must be distinguished from true electromyographic activity. Noise and artifacts associated with the use of bipolar electrocautery generate high-amplitude discharges that are easily distinguishable from genuine electromyographic activity. Continuous saline irrigation during surgery did not induce electromyographic activity (except in one case with dural sac rupture).

Reasons for the absence of intraoperative alarm activity with the potential for postoperative nerve injury (19) include: Complete and orderly nerve transection. Ischemic injury to the nerves rather than traction injuries to nerve roots. Traction injury to nerves as they move with limb manipulation. Unipolar or bipolar electrocoagulation burns masked by interfering waves. This necessitates continuous monitoring by neurophysiologists to observe changes in free EMG throughout surgery, not overlooking any abnormal EMG changes, and promptly notifying the surgical team.

In summary, intraoperative free EMG is a simple and feasible technique with relatively high sensitivity and specificity, helping reduce the risk of intraoperative nerve injury during total intravenous anesthesia UBE procedures. However, given the limited number of cases in this study, further large-sample, multicenter research is necessary to confirm the scientific validity and effectiveness of this method.

## Data Availability

The original contributions presented in the study are included in the article/Supplementary Material, further inquiries can be directed to the corresponding author.
